# Bridging Gaps in Diabetic Nephropathy Care: A Narrative Review Guided by the Lived Experiences of Patient Partners

**DOI:** 10.1177/20543581221127940

**Published:** 2022-10-11

**Authors:** William Beaubien-Souligny, Simon Leclerc, Nancy Verdin, Rizwana Ramzanali, Danielle E. Fox

**Affiliations:** 1 Division of Nephrology, Centre Hospitalier de l’Université de Montréal, QC, Canada; 2 Department of Medicine, University of Montreal, QC, Canada; 3 Division of Nephrology, Department of Medicine, The Research Institute of the McGill University Health Centre, Montreal, QC, Canada; 4 Division of Nephrology, Hôpital Maisonneuve-Rosemont, Montreal, QC, Canada; 5 The Kidney Foundation of Canada, London, ON, Canada; 6 Patient and Community Engagement Research Program, University of Calgary, AB, Canada; 7 Department of Community Health Sciences, University of Calgary, AB, Canada

**Keywords:** diabetic nephropathy, diabetes, kidney disease, screening, self-management, patient engagement

## Abstract

**Purpose of review:**

Diabetes affects almost a 10th of the Canadian population, and diabetic nephropathy is one of its main complications. It remains a leading cause of kidney failure despite the availability of effective treatments.

**Sources of information:**

The sources of information are iterative discussions between health care professionals and patient partners and literature collected through the search of multiple databases.

**Methods:**

Major pitfalls related to optimal diabetic nephropathy care were identified through discussions between patient partners and clinician researchers. We identified underlying factors that were common between pitfalls. We then conducted a narrative review of strategies to overcome them, with a focus on Canadian initiatives.

**Key findings:**

We identified 5 pitfalls along the diabetic nephropathy trajectory, including a delay in diabetes diagnosis, suboptimal glycemic control, delay in the detection of kidney involvement, suboptimal kidney protection, and deficient management of advanced chronic kidney disease. Several innovative care models and approaches have been proposed to address these pitfalls; however, they are not consistently applied. To improve diabetic nephropathy care in Canada, we recommend focusing initiatives on improving awareness of diabetic nephropathy, improving access to timely evidence-based care, fostering inclusive patient-centered care environment, and generating new evidence that supports complex disease management. It is imperative that patients and their families are included at the center of these initiatives.

**Limitations:**

This review was limited to research published in peer-reviewed journals. We did not perform a systematic review of the literature; we included articles that were relevant to the major pitfalls identified by our patient partners. Study quality was also not formally assessed. The combination of these factors limits the scope of our conclusions.

## Abrégé

**Motif de la revue:**

Le diabète touche près d’un dixième de la population canadienne et la néphropathie diabétique est l’une de ses principales complications. Le diabète demeure une cause principale d’insuffisance rénale malgré la disponibilité de traitements efficaces.

**Sources:**

Discussions itératives entre des professionnels de la santé et des patients partenaires, ainsi que la documentation recueillie à la suite d’une recherche dans plusieurs bases de données.

**Méthodologie:**

Les principaux obstacles liés aux soins optimaux en néphropathie diabétique ont été définis grâce à des discussions entre des patients partenaires et des cliniciens-chercheurs. Des facteurs sous-jacents, communs à tous ces obstacles, ont été dégagés, puis nous avons procédé à un examen narratif des stratégies visant à surmonter ces obstacles, en privilégiant les initiatives canadiennes.

**Principaux résultats:**

Cinq obstacles jalonnant la trajectoire de la néphropathie diabétique ont été identifiés, soit un retard dans le diagnostic du diabète, une régulation glycémique sous-optimale, un retard dans la détection de l’atteinte rénale, une protection rénale sous-optimale et une gestion déficiente de l’insuffisance rénale chronique de stade avancé. Plusieurs approches et modèles de soins novateurs ont été proposés pour remédier à ces obstacles, mais ils ne sont pas appliqués de façon uniforme. Pour améliorer les soins de néphropathie diabétique au Canada, nous recommandons de concentrer les initiatives visant la sensibilisation à la néphropathie diabétique, l’amélioration de l’accès en temps opportun à des soins fondés sur des données probantes, la promotion d’un environnement de soins inclusif axé sur le patient et la production de données probantes appuyant la gestion complexe de la maladie. Il est impératif que les patients et leurs familles soient au cœur de ces initiatives.

**Limites:**

Notre revue s’est limitée aux articles publiés dans des revues examinées par des pairs. Nous n’avons pas procédé à une revue systématique de la littérature; nous avons inclus des articles pertinents pour les principaux obstacles identifiés par nos patients partenaires. La qualité des études n’a pas été évaluée officiellement. La combinaison de ces facteurs limite la portée de nos conclusions.

## What was known before

Diabetic nephropathy is one of the main complications of diabetes. It remains a leading cause of kidney failure even if effective interventions are available.

## What this adds

There are multiple pitfalls along patient care trajectories that may prevent patients from benefiting from the recent improvements in preventive interventions. Innovative interventions and care approaches have been developed to improve the care continuum for patients with diabetic nephropathy in Canada. However, the lack of accessibility to timely evidence-based care, breaks in care continuity, and difficulties in self-management remain important barriers to overcome.

## Introduction

Diabetes is a common disease and a major public health challenge. According to Diabetes Canada, 3.4 million Canadians representing 9.3% of the population had diabetes in 2015, and it is estimated that its prevalence will rise to 12% of the population by 2025.^1^ A common complication of diabetes is diabetic nephropathy, which is the most frequent cause of chronic kidney disease (CKD) in adults and the leading cause of kidney failure in Canada, resulting in significant individual-level and system-level impacts.^2,3^

Diabetic nephropathy can be preventable with early diagnosis and optimal clinical management. However, there are many pitfalls in the journey of a person living with diabetes that can result in suboptimal care and outcomes. In this review, patient partners and clinician researchers collaborated to identify fundamental care gaps and approaches to care provision that aim to alleviate them.

## Methods

Our team comprised of 3 clinician researchers who work with people with diabetic nephropathy and 2 experienced patient partners: one living with diabetic nephropathy and one living with kidney failure who has been a peer mentor to individuals with this experience. Three additional patient partners were engaged throughout our inquiry to provide additional insight and feedback at specific moments.

**Figure 1.**
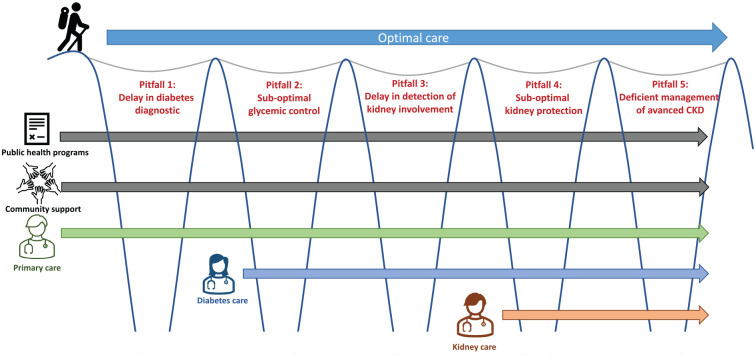
Pitfalls along the journey of the diabetic nephropathy. *Note.* CKD = chronic kidney disease.

Over a 4-month period, we engaged in iterative discussions with an aim of identifying major gaps in the care of people living with type 2 diabetes mellitus (T2DM) and diabetic nephropathy in Canada (Figure 1). We created a case vignette based on our collective experiences to outline a common, yet potentially preventable, trajectory from T2DM to kidney failure that illustrates common barriers to optimal care. The case vignette was then used to foster group discussions about pathways toward improvements and to guide a narrative review of research initiatives that aim to address these barriers. We searched OVID Medline, PubMed, and Google Scholar from inception to February 10, 2022. Keywords for the target population included “diabetes,” “diabetic nephropathy,” and “diabetes and kidney disease.” Keywords for the area of focus included “early/delayed/late diagnosis,” “screening,” “risk assessment/evaluation,” “self-management,” “self-care,” “self-efficacy,” “continuity,” “continuity of care,” “care coordination,” and “Canada.” A subset of peer-reviewed literature was selected from these searches as deemed relevant to our inquiry.

## Pitfalls Along the Journey of Diabetic Nephropathy

### Pitfall 1: Delay in Diabetes Diagnosis

*Jane had a fulfilling, yet hectic professional life as the manager of a very busy retail store. Dedicated to her family, friends, and community, she left little time for herself. When she started to feel fatigued, experience blurred vision, and feel an insatiable thirst, she searched online to try and find a health care provider who could see her. After a 2-week search, she was finally able to secure an appointment at an emergency clinic nearby. If only finding a family physician in her remote community was easier. The physician examined her but didn’t have much to say. A week later, she received a call that changed her life forever: she had diabetes, and her blood sugar levels were extremely high.*

Timely diagnosis of T2DM has a critical impact on the trajectory of diabetic nephropathy, and appropriate interventions implemented at this time mitigate its future impact on patients’ lives. Efficient interventions to prevent TD2M onset exist (eg, lifestyle modifications can reduce the progression from pre-diabetes to diabetes),^4,5^ making screening and early intervention important aspects of care provision. Although screening tests for diabetes are available, not every Canadian has access to a primary care provider (PCP) to order them. This may partially explain why up to 41% of Canadians that have T2DM are not diagnosed^6^ and why individuals typically wait 4 to 7 years before their diagnosis, which often only occurs after seeking urgent medical care for a severe complication.^7,8^ This lengthy delay represents a time where the patient is not treated and is a fertile ground for the various complications of diabetes to arise.

One avenue to improve access to timely screening is to ensure Canadians have access to trained PCPs. This may be especially true in rural jurisdictions in Canada, where a lack of medical practitioners remains a major barrier to equitable access to health services.^9,10^ The College of Family Physicians of Canada and the Society of Rural Physicians of Canada have recently implemented a roadmap for action, focused on improving access to health services in rural jurisdictions.^9^ Although most of these action items are in development phases, pilot programs have begun to show promising results.^9^ For example, new models of family medicine training that integrate regional campuses outside of metropolitan areas are increasing the likelihood that trainees will choose to practice rural family medicine upon graduation.^10,11^

There is evidence to suggest that current screening for diabetes in Canada is inadequate. For example, a recent study showed that 20% of individuals recently diagnosed with diabetes in Ontario had a glycated hemoglobin (HbA1C) higher than 8%, highlighting insufficient screening and delay in diagnosis.^12^ Society-wide screening programs have been proposed as a potential solution to this problem. However, vast screening programs are very difficult to organize and costly to implement. For example, European studies have shown that as few as 20% of people undergo their first screening test in such programs.^13^ For screening to be efficient and cost-effective,^14^ Diabetes Canada thus recommends screening adults over 40 years old who have risk factors for T2DM or diabetes-related conditions.

One other alternative is to explore mechanisms that remove the initial need for health care providers and/or prioritize health care provider referrals. For example, various forms of self-screening, sent by mail or available at self-service in public spaces, could provide a means for individuals to self-screen. Research in diabetes technology is quickly expanding, with artificial intelligence being frequently adopted to support diabetes management.^15,16^ New technologies, like mobile applications or websites using artificial intelligence to assess a patient’s need for screening or primary care appointments, have been used by some researchers for early disease detection and to support self-management techniques.^17^ These technologies may also help to prioritize the need for primary care support, wherein individuals at high risk could be subsequently flagged and linked with a health care provider. These interventions could also be leveraged by health care providers to facilitate shared decision-making and provide evidence to support testing, screening, and timely disease detection.

### Pitfall 2: Suboptimal Glycemic Control

*Jane was determined. The emergency physician transferred her to a primary care physician, and she was listening to all their tips. She wanted to beat the disease and feel better. Sometimes, the recommendations were puzzling. Eat less of this and more of that . . . the doctor often talked about ingredients she didn’t even know. She was warned that diabetes was dangerous for her heart and eyes, so she put all her efforts in achieving glycemic control. However, checking sugars, eating regularly, and figuring out pills are not easy when you are managing a store and raising 2 children. All of this meant that, despite her best effort, things were not perfect. She was fearful to ask for further advice, as she felt judged by her physician.*

Optimizing glycemic control is critical to prevent diabetic nephropathy and other related complications. However, glycemic management can be an incredible challenge for many people with diabetes. Diabetes self-management education and lifestyle modification programs are common interventions that focus on self-care and increasing knowledge and skill development.^18,19^ Strategies to build self-efficacy and behavior change are often incorporated into these programs.^19^ Lifestyle modification programs that focus on reducing risk factors for diabetes (eg, maintaining a normal body mass index, smoking cessation, blood pressure control, cholesterol control, optimizing physical activity) have particular utility early in the trajectory.^20^ As the structure and content of these programs are highly variable, it remains difficult to assess the extent of their impact, especially on long-term outcomes like disease progression.^19^ An array of technology to support diabetes management has also been applied. Technology-supported diabetes self-management was often most effective when combined with interactive data sharing, where feedback and effective communication and support by the health care team were simultaneously provided.^21^ Videogames and gamification have also been shown to be helpful tools to increase motivation and provide positive reinforcement in diabetes education and management.^22^

For self-management to be effective, large scale social programs to mitigate common self-management barriers need to be in place. Despite the known benefits of self-management and its importance in the prevention of diabetic nephropathy, many individuals are unable to partake in self-management initiatives. Self-management interventions pose an additional time commitment that many are unable to meet.^23,24^ Many individuals are further unable to implement suggested strategies as they can cause emotional and financial burden.^25^ Behavior change is complex, and individuals may experience difficulty coping with their disease which may limit their ability to participate in self-management.^26^ Many self-management services are also not covered by health care programs, and when they are, patients may still incur a significant cost burden in implementing recommendations (eg, purchasing more expensive diet friendly food, cost of traveling to appointments, private counseling, medication co-pay, and cost of diabetes management supplies).

Self-management interventions may further be inaccessible when they are not individualized.^27-29^ For example, many interventions do not account for nutrition and health practices across cultures or accommodate different languages and spiritual beliefs.^27^ Implementing standardized programs may not provide the individual with the knowledge and skills they require to address their unique self-management barriers. They may also not be of interest to the individual, and thus, they may choose not to engage in the initiative.^30^ A breadth of self-management interventions that can be customized to facilitate patient choice to foster more meaningful self-management is thus required.^24,27^

Effective self-management could be further complicated by negative relationships with health care providers wherein patients may feel unsupported and a lack of trust toward their health care provider.^25^ The creation of culturally appropriate support and safe environments is critical. For example, a cross-sectional study of 554 indigenous people in Ontario documented that indigenous people living with diabetes experienced barriers to culturally appropriate health services.^31^ This may partially explain why indigenous people in Ontario are less likely to have a family physician, report poor continuity of care, and are at higher risk for emergency department visit for dysglycemia.^32^

### Pitfall 3: Delay in Detection of Kidney Disease

*One day, Jane’s physician warned her that there was a new problem: proteins in her urine. How did this happen? She worked so hard to keep her blood sugars at her target, and what did proteins in the urine have to do with diabetes anyway? They explained to her that sometimes diabetes attacks the kidneys, but apart from the proteins, her kidney function was good. She was reassured, but it was a bit difficult to grasp . . . what role does the kidney have exactly? What were the risks for her in the long run? Why was this the first time she was hearing about this?*

The presence of kidney disease is often under-recognized by PCPs, which can have detrimental impact to kidney disease progression.^33^ Current screening tests (eg, creatinine, estimated glomerular filtration rate, and albuminuria) to detect kidney involvement are useful to raise the flag once kidney damage begins to occur and to initiate treatments to optimize kidney protection. However, low physician adherence to clinical guidelines for diabetes management in CKD is a concern in Canada, including the insufficient use of urine albuminuria testing and other screening mechanisms and inadequate use of recommended therapies.^34^

Risk prediction algorithms may support health care providers to identify those at risk for kidney disease and to support appropriate screening, treatment, and nephrology referrals.^33^ The Kidney Failure Risk Equation has been validated in the Canadian context for people with diabetes.^35,36^ It is also available freely online and may be accessed by patients and their families to support self-management. The kidney failure risk equation, however, has been more frequently used and validated for later stages of kidney disease, wherein its utility and widespread use among PCPs to support early disease detection and treatment in Canada have not been well elicited. Leveraging the capabilities of electronic medical records may also be an avenue to further improve process adherence.^37^ The integration of decision support tools into electronic medical records may support disease management by alerting health care providers to drug interactions, facilitate communication between multiple providers and patients, and optimize the tracking of disease progression without over-testing.^37^ In the context of T2DM and CKD, the electronic medical record could be set up with automated algorithms that could routinely order testing based on recommended guidelines and could integrate resources that support treatment decision-making.

In a recent Canadian survey, only 51.5% of individuals with T2DM and CKD were aware of their CKD diagnosis,^34^ emphasizing the critical need to engage patients in their care. Kidney Check Point-of-Care Testing is a Canadian screening initiative that works with patient partners to implement culturally safe strategies that target the early identification of kidney disease, risk stratification, and timely access to interventions in remote indigenous communities.^38^ The scale and spread of similar models of care may serve to fill CKD screening gaps for people with T2DM in Canada.

People with diabetes are likely to have had multiple and diverse encounters across health care professionals (eg, primary care, endocrinology, nephrology, and cardiology) and health care settings (eg, primary care clinics, specialty clinics, emergency and urgent care centers, and walk-in clinics) by the time kidney involvement occurs. A retrospective study in Ontario revealed that patients presenting with hyperglycemia in the emergency department who were then referred to specialized diabetes care had better short-term outcomes.^39^ Each encounter should thus be seen as an opportunity to evaluate risk factors of worsening disease and suboptimal disease management and to intervene with appropriate coordinated services where required. This includes opportunities to screen not only for risk factors for kidney disease but also for other related conditions (eg, cardiovascular disease and neuropathy). This may be especially pertinent when those who have been lost to follow up re-enter the health care system, provided that continuity of care can be achieved from this point on.

### Pitfall 4: Suboptimal Kidney Protection

*Jane’s doctor proposed treatments to protect her kidneys, and she accepted them with gratitude. However, she was prone to low blood pressure, and the new drugs made her feel dizzy and weak. Her physician even said that one of them had been toxic for her kidneys! She didn’t want anything to do with that. Luckily, she was told that her kidney function went back to normal after she stopped her new medications. Reassured, she continued with her regular diabetes medication.*

Suboptimal kidney protection results in faster progression of kidney disease for people with diabetes. However, managing multiple and often conflicting conditions creates significant challenges. Collaboration between health care providers both within and between clinics is required. Although important across the trajectory, this becomes especially pertinent once kidney involvement occurs, as potentially 3 or more speciality areas become integral to care (eg, endocrinology, nephrology, cardiology, PCPs, and other specialties). It is often unclear how responsibilities regarding the diverse tasks involved in the optimal follow-up of individuals are distributed among different health professionals and specialists.^40^ A recent population-based study in Ontario showed that continuity of care was lower in people with multimorbid diabetes, especially when comorbidities not related to diabetes are present.^41^ Difficulties in establishing effective communication strategies between professionals are often reported by all parties and may contribute to breaks in the continuity of care through divestment of either the PCP or the specialists.^42^ Digital platforms may facilitate the process of referral and ongoing communication between these actors. Preliminary experiences of these systems suggest a high satisfaction with timely response being a key facilitator between PCPs and nephrologists.^43^ Collaborative care agreements outlining the respective responsibilities of each party could facilitate this process to avoid care gaps.^44^

Optimal kidney protection relies not only on the coordination of care providers but also on accessible care. Strict adherence to guidelines in terms of the frequency of in-person follow-up may have negative consequences, particularly when conciliation between the management of health issues may conflict with other aspects of life. Decisions about how and when care is accessed must occur with the individual, and flexible care delivery models to support these decisions should be developed and implemented. Virtual care may be one of these areas as it offers opportunities to support easier access to health care teams and may be especially pertinent for those who become burdened by frequent appointments or who have accessibility challenges.^19,45^ Although virtual care has shown numerous benefits, identifying how to optimize its use is a priority, as it is not appropriate in all circumstances.^46,47^ This may be especially true in later stages of kidney disease where the patient’s physical evaluation is crucial to appropriately assess volume status and uremic symptoms.

Regardless of how care is coordinated and delivered, the improved medical management of diabetic nephropathy is essential. Suboptimal kidney protection is often hindered by complex polypharmacy concerns, changing pharmacokinetics, and variability in provider practices. One concern is the use of many nephrotoxic medications remaining prevalent despite their avoidance being recommended in national and international practice guidelines. For example, it has been shown that despite practice guidelines recommending the avoidance of non-steroidal anti-inflammatory (NSAID) medication in CKD, NSAID prescriptions/use remains relatively high.^48^ Education is also needed on how to prescribe and follow up with agents that optimize kidney protection, especially for agents such as RAAS and SGLT2i. Many health care providers may be unaware that these agents represent the cornerstone of kidney protection.^49^ Furthermore, they may be more likely to stop them permanently when a moderate increase in serum creatinine expected to normally occur with both RASS^50^ and SGLT-2i^51^ occurs or in the setting of moderate hyperkalemia which could be managed with pharmacological or dietary interventions.^52^ Individuals and health care providers often report difficulty navigating concerns with polypharmacy, medication burden, and medication cost. The financial burden of polypharmacy must not be understated. Not all Canadians have full coverage for their medications (as well as other elements of their care). For individuals that do, newer agents are often inadequately covered by provincial drug plans. For example, even if it is now well-known that the kidney protection conferred by SGLT2i is not mediated through improvement in glycemic control,^53^ coverage in some provinces still requires demonstrating suboptimal glycemic targets along with the prescription of other hypoglycemic agents. Advocacy at the provincial level is of paramount importance to make sure access to nephroprotective treatment evolves in tandem with new evidence.

Understanding how the patient responds to and views treatment interventions, engaging in shared decision-making, and supporting the self-management of these complex regimens is critical. Self-management interventions focused on addressing complexity and uncertainty are needed. Building problem-solving capacity may be one way to support people to better tackle the frequent complex problems they will encounter.^19^ Pharmacist-led interventions may also be effective at this stage given the challenges with medication management.^54^ In collaboration with other health care providers, pharmacists may be optimally placed to engage in shared decision-making, optimize and individualize treatment regimens, and support individuals to access coverage. Unfortunately, specialized pharmacist care is often not accessible, and many programs rely on community pharmacists who may not have the knowledge or capacity to take on this important role.

### Pitfall 5: Deficient Management of Advanced CKD

*A year later, Jane started to feel weak, a feeling that reminded her of the beginning of her disease. Although her blood sugars were in range, her weakness kept increasing. Headaches, itchy skin, nausea, and vomiting . . . the days were long, and she felt incapable of going to work. After discussion with her colleagues and husband, she went to the hospital. She was horrified to learn that her kidneys were extremely weak; they had stopped working. Weren’t they fine at her last appointment? Why wasn’t she followed more closely? Everything became a quick blur. A strange, weird plastic catheter was installed in her neck, and she was plugged to what looked like a bizarre washing machine. She was on dialysis.*

*How did Jane end up here?*

It can be difficult to manage care for people who progress to kidney failure. Peer support and coaching with people who have lived experience of the disease offer an opportunity to support self-management.^19^ The support that peers can provide span beyond education, as peers are known to act as advocates, cultural translators, and mentors.^19^ Mental health support (eg, mindfulness and cognitive behavioral therapy) is also critical at this stage to support stress reduction, symptom management, and decrease emotional distress.^19^ However, varied forms of mental health support are often not covered by provincial health systems.

In Ontario, only about a third of people with kidney failure had high PCP continuity,^55^ indicating that follow-up is often entirely transferred to the care of the specialists at some point in their journey.^56^ Many PCPs or endocrinologists wrongly assume that kidney clinics have all the means and knowledge to assure all the patient’s needs are met. In reality, nephrologists are not trained to treat common ailments like depression and musculoskeletal pain, and kidney clinics or dialysis units often do not have the capacity to support the individual’s holistic needs. From a patient’s perspective, PCP and endocrinologist involvement remains generally appreciated by people with kidney failure.^57^ Although it may not influence overall survival or hospitalization risk,^55^ it is likely to alleviate care gaps related to health problems that are common in primary care.^58^

Interventions to improve continuity and collaboration between providers at this stage are important. One of the solutions would be to offer a different approach to PCP and diabetes follow-up to those with diabetes and kidney failure. An important care model to consider is shared-care clinics, which integrate health care providers from different specialties who collaborate to provide care to patients with complex medical issues at a single location.^59,60^ For example, many patients with T2DM also have CKD and cardiovascular disease which can complicate health care provision and disease management. The Cardiac and Renal Endocrine Clinic is a multidisciplinary and interdisciplinary clinic at the Toronto General Hospital in Ontario, Canada, wherein cardiologists, nephrologists, and endocrinologists develop a single management plan with the patient during a single consultation.^59^ Marked improvements in low-density lipoprotein cholesterol, HbA1C, and blood pressure and a higher uptake of evidence-based medication were noted among patients.^59^ Lessons from care models applied earlier in the trajectory may have utility in structuring care delivery mechanisms across all disease stages. The diabetes empowerment group program is a pilot effort led by McGill University in Montreal, Canada. Group sessions are facilitated by physicians and nurses, include knowledge translation activities, and foster patient involvement as active partners.^61,62^

While alternative models of care delivery including community-based care, self-management programs, peer coaching, nurse-led community clinics, and shared-care clinics^63-67^ have been reported across the trajectory, it is unlikely that these are widely available. As such, expanding models of care provision across jurisdictions that can better meet the capacity, interest, and support needs of individuals with diabetic nephropathy may be an interesting approach to targeted complex care provision.^67^

## Perspectives on the Barriers to Optimal Diabetic Nephropathy Care

We identified 5 pitfalls along the diabetic nephropathy trajectory, including a delay in diabetes diagnosis, suboptimal glycemic control, delay in the detection of kidney involvement, suboptimal kidney protection, and deficient management of advanced CKD (Figure 1).

**Figure 2.**
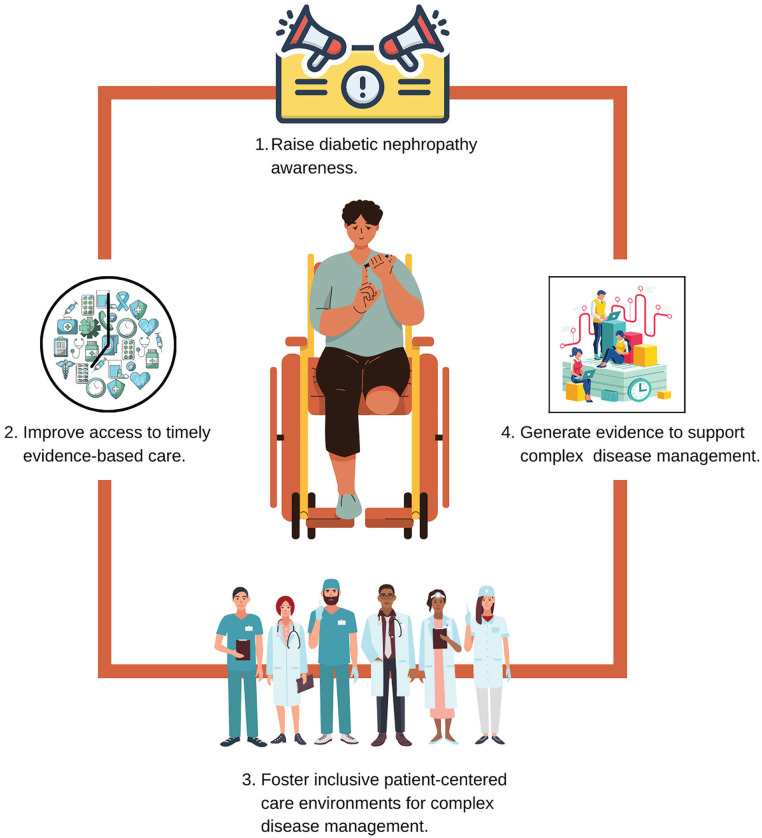
Improving diabetic nephropathy care in Canada.

This review was limited to research published in peer-reviewed journals. We did not perform a systematic review of the literature or explore all the possible root causes and promising interventions to improve diabetic nephropathy care. Data quality was not formally assessed nor were meta-analyses conducted to determine the overall effect of the evidence discussed. The combination of these factors limits the scope of our conclusions. However, our narrative review was founded from our collective lived experiences and knowledge of the literature, which led us to identify 4 areas that we believe should be better understood and targeted to improve care provision in this domain (depicted in Figure 2).

### Raise Diabetic Nephropathy Awareness

Awareness and early intervention are critical to prevent diabetic nephropathy and support effective T2DM care provision. Most individuals with T2DM are unaware that diabetes can lead to kidney disease, and many with T2DM and CKD are not aware that they have kidney disease. Health care providers and patients may also not be aware of or implement best practice guidelines or know how to effectively manage agents that optimize kidney protection. The lack of physical symptoms of kidney involvement until late-stage disease makes it easy to be overlooked. This problem thus extends beyond creating better access to care and an awareness of the possibility of kidney disease to understanding and emphasizing the implications and complications that it can create for a person living with diabetes early in the trajectory. The community must wake people up to the realities of kidney involvement and how detrimental its impact can be and promote early awareness, disease detection, and evidence-based treatment.

### Improve Access to Timely Evidence-Based Care

It takes significant coordination to effectively manage T2DM and diabetic nephropathy. Timely and appropriate access to health care is a prerequisite to appropriate diagnosis and treatment. Despite continuous efforts to assure that every Canadian has access to appropriate health care provisions, barriers in accessing health care providers, timely screening, and evidence-based treatment remain. In many jurisdictions, access to health care providers and needed services are often limited. Those with access to a health care provider may still face poor continuity of care and rely on health care providers who may lack confidence in their knowledge of appropriate recommendations and treatments to prevent disease occurrence and progression. Suboptimal care coordination and individual and social barriers to accessing care and self-management interventions further hinder access to appropriate treatment and can make knowledge acquisition and self-management difficult.

### Foster Inclusive Patient-Centered Care Environments for Complex Disease Management

Few health care models incorporate holistic complex disease management across the disease trajectory. Improper orchestration of health care professionals, a focus on downstream interventions, and health care models that may not be structured to adapt to the needs of the individual contribute to this problem. Care coordination founded in empathy, strong communication, and equal partnership between all players is needed to build trust and effective care provision. Ultimately, the patient knows their body best. They know what works and what does not, and they should be considered the greatest resource in both understanding the disease itself and how it can be managed most effectively. Current biomedical models of health care delivery largely place the power in the hands of health care professionals to coordinate care and determine testing, treatment, and self-management options. Opportunities to shift this power to the patient and their family are an important avenue forward.

### Generate New Evidence to Support Complex Disease Management in Canada

To overcome the multifaceted problems that surround diabetic nephropathy care, innovative solutions are required. Integrated research, clinical, and quality improvement models that span across silos and that are founded in the patient experience may serve to generate meaningful evidence and a way forward. These models should include patient experiences that are diverse and represent multiple views and experiences. The research community should thus focus on continuing to establish systems where patients can not only partner in this work but also lead it. The closer integration of clinical and research centers may not only facilitate this process but provide mutual benefit. For example, clinicians and patients may benefit from timely access to evidence, and researchers would have improved access to supportive study sites and potential participants. Established relationships and collaboration from all parties may foster research initiatives that are meaningful to all.

Discussions between patients, researchers, and policymakers should further be supported by evidence adapted to the Canadian setting. This involves prioritizing public health research investigating the net impact of strategies aiming to reduce the burden of diabetic nephropathy in Canada. Awareness and exposure of knowledge end-users about existing evidence and the innovative work that is already occurring in Canada may support its application. Research into therapies and care environments that support complex care must recognize the many interrelating factors that impact health in this population and focus on complex interventions, treatments, and systems that facilitate safe and effective multi-morbidity management.

## Conclusions

Diabetic nephropathy is a major cause of morbidity and mortality for people with diabetes and represents one of the leading causes of kidney failure in Canada. Although our knowledge of the disease is improving, there remain many pitfalls that prevent effective care provision and disease management. Working toward closing these gaps and translating research results into practice should be the priority of everyone involved in the care of people with, and at risk for, diabetic nephropathy.
